# Effects of Stance Width and Barbell Placement on Kinematics, Kinetics, and Myoelectric Activity in Back Squats

**DOI:** 10.3389/fspor.2021.719013

**Published:** 2021-09-01

**Authors:** Stian Larsen, Eirik Kristiansen, Eric Helms, Roland van den Tillaar

**Affiliations:** ^1^Department of Sports Sciences and Physical Education, Nord University, Levanger, Norway; ^2^Sports Performance Research Institute New Zealand, Auckland University of Technology, Auckland, New Zealand

**Keywords:** sticking region, strength, powerlifting, electromyography, squat, inverse dynamics

## Abstract

Barbell placement and stance width both affect lifting performance in the back squat around the sticking region. However, little is known about how these squat conditions separately could affect the lifting performance. Therefore, this study investigated the effects of stance width and barbell placement upon kinematics, kinetics, and myoelectric activity around the sticking region during a three-repetition maximum back squat. Nine men and nine women (body mass: 76.2 ±11.1, age: 24.9 ± 2.6) performed back squats with four different techniques, such as: high-bar narrow stance (HBNS), high-bar wide stance, low-bar narrow stance, and low-bar wide stance where they lifted 99.2 ± 23.6, 92.9 ± 23.6, 102.5 ± 24.7, and 97.1 ± 25.6 kg, respectively. The main findings were that squatting with a low-bar wide stance condition resulted in larger hip contributions to the total moment than the other squat conditions, whereas squatting with an HBNS resulted in greater knee contributions to the total moment together with higher vastus lateralis and less gluteus maximus myoelectric activity. Our findings suggest that training with an HBNS could be beneficial when targeting the knee extensors and plantar flexors, whereas a low-bar wide stance could be beneficial when targeting the hip extensors.

## Introduction

When the goal is to strengthen the lower extremities, different variations of the back squat are frequently used in resistance training (van den Tillaar and Larsen, 2020). Several studies have demonstrated that kinematics and kinetics are affected by stance width and barbell placement (Glassbrook et al., 2017, 2019). Lahti et al. (2019) investigated the effect of wide and narrow stance width upon joint kinematics, and sagittal and frontal joint moments on the hip and knees on 14 amateur rugby players at 70 and 85% of 1 repetition maximum (1-RM) in the back squat. The investigators found greater hip flexion and abduction for the wide stance width. In comparison, knee flexion was greater for the narrow stance width. When investigating the kinetics, they found that the wide stance width resulted in a greater hip extensor and knee adduction moment. In contrast, the narrow stance width resulted in a greater knee extensor moment at both 70 and 85% of 1-RM. A wide stance width has been shown in several studies to generate a different myoelectric activity profile than the narrow stance width in the back squat (Anderson et al., 1998; McCaw and Melose, 1999). Moreover, McCaw and Melose (1999) found that a wide stance width resulted in greater myoelectric activity in the adductor longus and gluteus maximus. Furthermore, as observed in earlier studies at maximal and near-maximal squat attempts, a sticking region occurs during the ascent (Escamilla et al., 2001; Maddox et al., 2020; Larsen et al., 2021b). This type of research often divides the ascent into three regions based on the velocity curve. The presticking region is the region between the lowest barbell height (*v*_0_) and the first peak in barbell velocity (*v*_max1_). The sticking region is the region between *v*_max1_ and the first local minimum barbell velocity (*v*_min_). Lastly, the poststicking region is the region between *v*_min_ and the second peak in barbell velocity (*v*_max2_) (Larsen et al., 2021b). It may therefore be of interest to athletes, practitioners, and coaches to know how squatting with different stance widths could manipulate the demands on the muscles and joint moments responsible for the sticking region because to our knowledge this has yet to be done.

The two most common back squat bar placements are the high-bar and the low-bar placement, characterized by the bar placed along the top of the upper trapezius or across the midtrapezius, over the spine of the scapulae and posterior deltoid, respectively (Glassbrook et al., 2017). Several studies have investigated the effect of barbell placements on squat performance and biomechanics (Benz and West Chester, 1989; Fry et al., 1993; Wretenberg et al., 1996; Swinton et al., 2012; Glassbrook et al., 2017, 2019). Furthermore, when squatting with loads corresponding to greater than 85% of 1-RM, a sticking region has been observed (Elliott et al., 1989). Only one study investigated the effect of barbell placement on kinematics and myoelectric activity specifically during the sticking region (van den Tillaar et al., 2020). van den Tillaar et al. (2020) investigated the effect of barbell placement upon kinematics and myoelectric activity when the barbell load was matched between the squat conditions and found no significant differences in barbell velocity, displacement, or joint angles at any of the time point “events” in and around the sticking region between the low-bar and the high-bar back squat. Moreover, they observed greater myoelectric activity for the rectus femoris, vastus medialis, and lower part of erector spinae for the high-bar position. In the sticking region, the gluteus medius and maximus myoelectric activity increased for both barbell placements, whereas myoelectric activity decreased in the quadriceps and soleus muscles. Also, the low-bar squat can result in a more anterior projection of the center of mass than the high-bar squat (Swinton et al., 2012). This may be explained by the observation that squatting with a low-bar technique often leads to greater forward lean and thereby a greater horizontal distance between the barbell and the hip joint, creating greater external hip joint moments and moment arms as speculated by van den Tillaar and Larsen (2020).

The low-bar squat is often characterized by a wider stance width and used by powerlifters, whereas the high-bar squat is often characterized by a narrow stance width and used by weightlifters (Glassbrook et al., 2019). However, to the knowledge of the authors, no works have investigated the combined effect of both stance width and barbell placement upon kinematics, kinetics, and myoelectric activity around the sticking region for different squat conditions. Therefore, this study aimed to investigate the effects of stance width and barbell placement upon the kinematics, kinetics, and myoelectric activity around the sticking region during a three-repetition maximum back squat. It was hypothesized that the low-bar conditions would produce greater hip contributions than the high-bar conditions independent of stance width, creating increased demand on the hip extensor muscles and the possibility for lifting greater loads because the low-bar squat, typically in the literature, is referred to as more hip dominant than the high-bar squat (Glassbrook et al., 2017). Also, it was hypothesized that hip moment arms and hip contribution to the total moment would peak in the sticking region independent of the squat condition.

## Methods

### Experimental Approach to the Research Question

To investigate the effect of stance width and barbell placement on kinematics, kinetics, and myoelectric activity around the sticking region, a within-subjects, repeated measures design was used. Two stance widths (narrow and wide) and two barbell placements (high-bar and low-bar) were used. This resulted in four squat conditions, such as: high-bar narrow stance (HBNS), high-bar wide stance (HBWS), low-bar narrow stance (LBNS), and low-bar wide stance (LBWS) as independent variables. Dependent variables included mean myoelectric activity during the presticking, sticking, and poststicking regions, as well as net joint moments and moment arms, ground reaction forces, joint angles, barbell velocity, time, and displacement in the events *v*_0_, *v*_max1_, peak barbell deacceleration (*d*_max1_), *v*_min_, and *v*_max2_.

### Participants

Eighteen participants who were recreationally trained lifters volunteered for this study (Table 1). Inclusion criteria were: (1) the men had to be able to lift at least 1.5 times their body mass, and women one time their own body mass in 1-RM for the preferred squat condition since men are reported to elicit greater maximal absolute strength in the lower extremity (Bishop et al., 1987); (2) none of the participants could have an injury or illness that could influence the maximal performance on the test; (3) participants had to perform the depth requirement set by the International Powerlifting Federation (IP Federation, 2019) for all squat conditions, which was that the top surface at the hip joint was below the knees in the bottom position when viewed laterally; (4) participants had to perform three familiarization sessions and two tests to be included in the analysis to ensure that they were familiar with performing all squat conditions and that the proper 3-RM was achieved in every squat condition. Written consent was obtained from all the participants before participation. The study was conducted in accordance with the latest revision of the Declaration of Helsinki and current ethical regulations for research and was approved by the National Center for Research Data (pr.nr: 701688).

**Table 1:** Mean ± SD characteristics and anthropometrics of the participants.

**Physical property**	**Mean ± SD**	**Range**	**Male**	**Female**
Age (years)	24.9 ± 2.6	22–30	26.4 ± 2.7	23.6 ± 1.5
Height (cm)	173 ± 8.6	160–186	180.1 ± 6.8	167 ± 3.6
Weight (kg)	76.2 ± 11.1	59.6–92.4	83.9 ± 8.6	69.3 ± 8.4
Fat percentage (%)	21.8 ± 5.3	12.9–31	17.7 ± 2.6	25.4 ± 4.4
Distance from c7 low-bar (cm)	7.4 ± 1.8	5.0–10.8	8.5 ± 1.8	6.6 ± 1.3
Stance width narrow (cm)	31.7 ± 2.7	27.6–37.0	33.7 ± 2.3	29.9 ± 1.5
Stance width wide (cm)	59.8 ± 5.1	52.2–69.9	63.6 ± 4.4	56.4 ± 2.8

### Procedures

All participants participated in three familiarization sessions and two test sessions. The participants were given augmented feedback regarding the technique from two experienced powerlifting coaches during the familiarization sessions to ensure proper performance during test days. To prevent unnecessary exhaustion that could impact the performance, the participants had a minimum of 4 days of rest between the familiarization sessions and 7 days of rest between the test sessions.

On familiarization day one, the participants completed a questionnaire reporting their previous 1-RM and preferred stance width and signed the consent form. Thereafter height, body mass, and fat percentage were measured. Body mass and fat percentage were measured on a Tanita scale (MC-780MA, Riga, Latvia). Acromion length was measured horizontally from the right to the left acromion to decide the narrow and wide stance width for every participant, where 0.7 times the horizontal acromion length was used as a narrow stance and 1.7 times the horizontal acromion length was used as a wide stance. The stance width was marked with tape and was kept similar throughout the entire study. The participants needed to stand on the tape with the medial part of the calcaneus during the 3-RM tests in both the familiarization and test sessions. The required squat depth was marked with a horizontal band which was standardized and used for all familiarization and test sessions such that the proximal part of the hamstring had to touch the horizontal band before starting the ascent. The barbell placement for the low-bar was measured as the axial distance from the spinous process of the vertebra to the barbell (low-bar: 7.4 ± 1.8 cm). In the first familiarization session, each participant squatted up to three repetitions with 60% of predicted 3-RM with the self-reported preferred barbell placement. During familiarization sessions two and three, participants tested a 3-RM for each of the remaining squat conditions which were not tested during the first familiarization session. 3-RM was used since it is a typical load used in training for increasing maximal strength among powerlifters. No additional guidance on how to perform the different stance widths was given. The order for the squat conditions during the familiarization and test sessions were randomized on www.randomizer.org. During familiarization 3-RM testing, the repetitions in reserve-based rating of perceived exertion scale (Zourdos et al., 2016) and mean concentric barbell velocity were utilized to enhance testing accuracy. Specifically, mean concentric barbell velocity of the final repetition was recorded during each 3-RM test to ensure similar last-rep 3-RM velocities in each testing condition to ensure that true maximums were achieved. Participants had 180 s of rest between warm-up sets and 240 s of rest between test sets during all testing sessions.

On test days, electrodes for electromyography (EMG) measurements and reflective markers for the motion capture measurements were attached to the body. After a general warm-up, which included three sets of 6–10 repetitions with an unloaded Olympic barbell (Rogue, Ohio power bar), the participants performed a standardized warm-up protocol with the first squat condition. The squat protocol was as follows: four repetitions with 40% of the lowest obtained familiarization 3-RM, three repetitions with 55% of the lowest obtained familiarization 3-RM, followed by three repetitions with 70% of the lowest obtained familiarization 3-RM. The first test set started at the lowest familiarization 3-RM to ensure that the participants did not fail due to fluctuations in daily readiness and strength (Greig et al., 2020; Larsen et al., 2021a). Thereafter, the load was increased from 1 to 10 kg based on the proximity to the mean concentric barbell velocity for the specific squat condition achieved in the familiarization session, or if the participant failed the third repetition. After completing each 3-RM squat condition, the participants started the next squat condition at the lowest obtained familiarization 3-RM.

### Recordings

A linear encoder (ET-Enc-02, Ergotest Technology AS, Langesund, Norway) was attached to the right side of the barbell to measure vertical barbell velocity and displacement with a resolution of 0.019 mm and 200 Hz sampling rate. The barbell velocity was calculated with a five-point differential filter using Musclelab (Musclelab version: 10.200.90.5095, Ergotest innovation, Porsgrund, Norway). On the third repetition of each squat condition, the vertical barbell velocity and displacement were calculated for the following events: *v*_0_, *v*_max1_, *d*_max1_, *v*_min_, and *v*_max2._ Vertical ascent displacement was measured from *v*_0_.

Musclelab (Musclelab version: 10.200.90.5095, Ergotest innovation, Porsgrund, Norway) was used to record EMG myoelectric activity of the following muscles on the dominant side of the participants: erector spinae iliocostalis, erector spinae longissimus, gluteus maximus, gluteus medius, semitendinosus, biceps femoris, adductor longus rectus femoris, vastus lateralis, vastus medialis, gastrocnemius medialis, and soleus medialis using SENIAM recommendations (Hermens et al., 2000) for location and orientation. The skin of the participants was shaved, scrubbed in alcohol, and dried with paper to reduce skin impendence before electrodes (11 mm contact diameter, 20 mm center to center distance). They were placed on the right side of the 12 muscles with a sampling rate of 1,000 Hz. Conductive gel (Signa Gel, Parker Laboratories INC, NJ, USA) was applied to the electrodes to reduce noise. Raw EMG signals were amplified and filtered with a preamplifier. These signals were high pass and low pass (500, 20 Hz) filtered. The raw EMG signals were converted to the root of mean square (RMS) signals with a hardware circuit network, which had a common rejection rate of 106 dB. The mean RMS was calculated for the presticking, sticking, and poststicking regions. For normalization, the participants performed a 5-s maximal voluntary isometric contraction (MVIC) squat at the same depth, barbell placement, and stance width as the bottom position performed with the HBNS where the barbell was mounted to a squat rack, which could be adjusted axially. The participants were instructed to obtain maximal force as quickly as possible and maintain the force throughout the trial. The mean RMS between 2.0 and 4.0 s was used as MVIC, whereas the mean RMS in the regions (presticking, sticking, and poststicking) was divided by the mean RMS between 2.0 and 4.0 s of the MVIC trial for normalization.

A three-dimensional motion capture system (Qualisys, Gothenburg, Sweden), with eight cameras at a sampling rate of 500 Hz and integrated force platforms, was used to track reflective markers and three-dimensional ground reaction forces. Markers were placed on both sides of the body, except for the upper and lower hand, where the markers were placed on the dominant side. Markers for the foot and shank were placed on the first and fifth proximal phalanx, the lateral and medial malleolus, and the femoral lateral and medial epicondyle. Markers for the pelvis were placed on the anterior superior iliac spine and posterior superior iliac spine, creating a coda pelvis and hip joint center (Bell et al., 1987, 1990). Markers for the thorax were placed on the acromion, C7 spinous process of the vertebra, TV1 thoracal process of the vertebra, the midpoint between the inferior angles of the most caudal points of the two scapulae, sternum jugular notch, and sternum xiphisternal joint (C-Motion, 2017). Markers for the upper and lower arm segment were placed on the medial and lateral epicondyle of the humerus and the radial and ulnar styloid process. Also, four markers were placed on the barbell with a 20-cm distance to track the events *v*_0_, *v*_max1_, *d*_max1_, *v*_min_, and *v*_max2_. Two force platforms (AMTI Multi-axis Force Transducer BP6001200-2000, Lexington, MA, USA; Kistler force platform, type 9260AA6, Winterthur, Switzerland) were integrated into the Qualisys motion capture system to track the three-dimensional ground reaction forces and enable inverse dynamics calculation. The origin of the axes was set to the corner of the left force platform. The *x, y*, and *z* axes were set to mediolateral, anterior–posterior, and vertical orientations, respectively. Mediolateral and anteroposterior forces were calculated because they could result in different directions of the ground reaction force vector and cause different sagittal and frontal moments. Due to negligible values of anteroposterior forces, they were not included in the analyses.

### Data Analysis

Motion capture data were exported to C3D files for segment modeling and analyses in Visual 3D v6 software (C-motion, Germantown, MD, USA). All computations from the model-based data were smoothed with a lowpass Butterworth filter at a cut-off frequency at 10 Hz. Joint angles for the torso, hip, knee, and ankle in the events *v*_0_, *v*_max1_, *d*_max1_, *v*_min_, and *v*_max2_ were calculated in the distal to proximal orientation with a Cardan sequence in the order *x*–*y*–z.

Joint angles for the hip, knee, and ankle were calculated as the angle between the distal and proximal segments, and the torso angle was calculated as the angle between the torso segment and the laboratory. The three-dimensional joint moments for the hip, knee, and ankle were calculated using inverse dynamics calculations in a resolute coordinate system. The joint moments calculated in this study are internal net joint moments, expressed as means and standard deviations at events *v*_0_, *v*_max1_, *d*_max1_, *v*_min_, and *v*_max2_ with respect to the resolute coordinate system of the distal segments. This was calculated to observe how the joint moments changed through the ascent events. The reported net joint moments data were summed between the right and left segments. Net joint moments from the sagittal plane are flexion and extension moments, and net joint moments from the frontal plane are abduction and adduction moments. Net joint moments from the analyzed planes were normalized to the mass of the participants using default normalization and expressed as Nm/kg. When calculating the hip, knee, and ankle contributions to the total net joint moments, all abduction and adduction values were normalized into positive values.

## Statistics

To assess differences between the sexes in the load lifted, an independent samples *t*-test was performed. For differences between sexes in barbell kinematics, a repeated 2 (sex: male, female) × 2 (stance width: narrow, wide) × 2 (barbell placement: high-bar, low-bar) × 5 (event: *v*_0_, *d*_max1_ *v*_max1_, *v*_min_, and *v*_max2_) analysis of variance was performed (ANOVA). To assess the difference in the load lifted and the joint angular velocities, together with their timings between the two stance widths and barbell placement, a repeated 2 (stance width: narrow, wide) × 2 (barbell placement: high-bar, low-bar) ANOVA (two-way ANOVA) was performed. For torso angles, ground reaction forces, net joint moments, moment contributions to total moment, and moment arms, a repeated 2 (stance width: narrow, wide) × 2 (barbell placement: high-bar, low-bar) × 5 (event: *v*_0_, *d*_max1_ *v*_max1_, *v*_min_, and *v*_max2_) ANOVA was performed. For myoelectric activity, a repeated 2 (stance width: narrow, wide) × 2 (barbell placement: high-bar, low-bar) × 3 (regions: presticking, sticking, and poststicking) ANOVA was performed. Bonferroni *post-hoc* tests were used to identify where potential differences in barbell kinematics, joint kinematics, myoelectric activity, and joint kinetics occurred. If the assumption of sphericity was violated, the Greenhouse–Geisser adjustments of *p*-values were reported. All results are presented as mean ± SDs. Effect sizes were evaluated with $\eta_{\text{p}}^{2}$ (partial eta squared), where < 0.01–0.06 constitutes a small effect, < 0.06–0.14 a medium effect, and > 0.14 a large effect (Cohen, 1988). The alpha level of significance was set at *p* < 0.05. Statistical analyses were conducted in SPSS version 27.0 (IBM Corp. Armonk, NY, USA).

### Results

A significant effect (*F* ≥ 28.86, *p* ≤ 0.001, η^2^ ≥ 0.51) of barbell placement and stance width was found for the load lifted, where squatting with a narrow stance width allowed for more load to be lifted (*p* ≤ 0.001). Moreover, squatting with a low-bar placement allowed the participants to squat with a greater load independent of stance width (*p* ≤ 0.001). Men lifted significantly more load (*p* ≤ 0.001) than women during all squat conditions, but followed the same pattern of loads lifted for the different squat conditions (see Table 2).

**Table 2:** Mean ± SD load lifted for all participants, males, and females during the high-bar narrow stance, high-bar wide stance, low-bar narrow stance, and low-bar wide stance during 3-RM back squats.

**Squat condition**	**All participants (kg)**	**Male (kg)**	**Females (kg)**
High-bar narrow stance	99.2 ± 23.6^‡^	118.6 ± 10.9^‡^	82 ± 17.3^‡^
High-bar wide stance	92.9 ± 23.6	112.7 ± 13.5	75.3 ± 14.6
Low-bar narrow stance	102.5.9 ± 24.7*	122.2 ± 10.1*	85 ± 19.9*
Low-bar wide stance	95.6 ± 25.4^†^	117.7 ± 16.7^†^	78.3 ± 15.9^†^

‡ *Indicates a significant difference in load lifted between the high-bar narrow stance and high-bar wide stance on a p ≤ 0.05 level*.

* *Indicates a significant difference in load lifted between the low-bar narrow stance and all other squat conditions on a p ≤ 0.05 level*.

† *Indicates a significant difference in load lifted between the low-bar wide stance and high-bar wide stance on a p ≤ 0.05 level*.

No significant effect (*F* ≤ 2.84, *p* ≥ 0.108, η^2^ ≤ 0.12) was found between sexes in barbell kinematics. Descent displacement was 0.64 ± 0.05, 0.6 ± 0.04, 0.63 ± 0.05, and 0.61 ± 0.05 m for the HBNS, HBWS, LBNS, and LBWS, respectively, whereas squatting with a narrow stance resulted in a greater descent displacement than the wide stance widths (*p* < 0.001; see Table 3).

**Table 3:** Mean ± SD hip, knee, and ankle angles for the high-bar narrow, high-bar wide, low-bar narrow, and low-bar wide squat conditions at the events *v*_0_, *v*_max1_, *v*_min_, and *v*_max2_ during the back squat at 3-RM.

**Event**	**Condition**	**Torso angle (°)**	**Hip flexion (°)**	**Hip abduction (°)**	**Hip external rotation (°)**	**Knee flexion (°)**	**Ankle plantar flexion (°)**
*v*_0_	High-bar narrow	46.7 ± 2.9	111 ± 7.2	−13.5 ± 8.6*	7.2 ± 5.5*	126.4 ± 4.7^‡^	106.0 ± 4.5^†^
	High-bar wide	46.0 ± 3.4	110.4 ± 4.5	−24.1 ± 7.0^†*^	15.7 ± 5.2^†*^	119.3 ± 6.4	98.6 ± 5.9
	Low-bar narrow	56.7 ± 2.5^‡^	110.5 ± 6.6	−11.5 ± 7.2	4.0 ± 5.9	122.7 ± 5.0^†^	106.3 ± 5.0^†^
	Low-bar wide	53.0 ± 4.4*	111.9 ± 4.8	−20.8 ± 8.9^†^	14.2 ± 6.4^†^	120.3 ± 5.8	100.5 ± 6.3
*v*_max1_	High-bar narrow	51.0 ± 3.6↓	106.8 ± 6.0↓	−9.4 ± 8.6↓^*^	7.1 ± 5.8*	114.7 ± 5.4^↓‡^	102.0 ± 4.1^↓†^
	High-bar wide	48.4 ± 2.8↓	108.7 ± 4.8↓	−20.6 ± 6.5^↓†*^	13.6 ± 5.2^†*^	111.1 ± 5.6↓	96.8 ± 5.4↓
	Low-bar narrow	61.0 ± 2.6^↓‡^	107.1 ± 6.6↓	−7.9 ± 6.5↓	4.6 ± 6.1	111.9 ± 6.2↓	103 ± 5.0^↓†^
	Low-bar wide	57.4 ± 5.2↓^*^	108.3 ± 6.1↓	−16.8 ± 8.5^↓†^	11.8 ± 5.7^†^	108.8 ± 5.3↓	97.4 ± 6.0↓
*d*_max1_	High-bar narrow	53.2 ± 2.8↓	100.5 ± 9.9↓	−7.0 ± 8.7*	5.6 ± 5.9↓	99.9 ± 9.2^↓‡^	97.3 ± 6.5^↓†^
	High-bar wide	52.3 ± 3.1↓	102.5 ± 7.2↓	−16.5 ± 6.5^†*^	9.8 ± 5.9^↓†^	96.1 ± 8.7↓	91.6 ± 6.3↓
	Low-bar narrow	63.5 ± 3.2^↓‡^	100.2 ± 9.6↓	−5.0 ± 6.6	3.7 ± 5.8↓	95.6 ± 9.9↓	97.4 ± 6.4^↓†^
	Low-bar wide	60.7 ± 4.8↓^*^	102.1 ± 9.1↓	−14.3 ± 8.1^†^	9.5 ± 6.0^↓†^	95.5 ± 6.5↓	93.1 ± 6.9↓
*v*_min_	High-bar narrow	54.5 ± 3.2	92.5 ± 9.1↓	−5.9 ± 8.1*	3.3 ± 4.7↓	88.1 ± 8.1^↓‡^	94.1 ± 6.2^↓†^
	High-bar wide	52.3 ± 4.9	96.1 ± 8.7↓	−15.0 ± 6.3^†*^	6.6 ± 5.7^↓†^	86.9 ± 8.7↓	89.6 ± 6.2↓
	Low-bar narrow	63.3 ± 5.0*	94.1 ± 10.2↓	−4.4 ± 6.3	2.3 ± 6.2↓	84.9 ± 5.6↓	93.8 ± 6.3^↓†^
	Low-bar wide	62.6 ± 7.4*	96.2 ± 10.1↓	−12.7 ± 7.0^†^	5.6 ± 5.7^↓†^	82.6 ± 4.9↓	89.4 ± 6.7↓
*v*_max2_	High-bar narrow	31.3 ± 3.2↓	46.5 ± 10.0↓	−7.0 ± 5.4	−9.8 ± 6.0↓	50.2 ± 5.7↓^*^	87.4 ± 5.0^↓†^
	High-bar wide	28.6 ± 4.4↓	48.7 ± 7.2↓	−14.6 ± 3.6^†^	−11.1 ± 6.5↓	50.1 ± 6.6↓^*^	83.7 ± 5.4↓
	Low-bar narrow	41.5 ± 4.9↓^*^	52.1 ± 9.5↓^*^	−5.7 ± 3.1^†^	−9.3 ± 6.8↓	48.3 ± 6.7↓	85.3 ± 5.0↓
	Low-bar wide	39.3 ± 4.5↓^*^	53.5 ± 7.9↓^*^	−12.7 ± 5.3^†^	−9.4 ± 7.0↓	47.0 ± 6.5↓	81.8 ± 5.2↓

*Torso angles are relative to a lab as a reference segment*.

↓ *Indicates a significant difference in torso angle between this event and all other events on a p ≤ 0.005 level*.

* *Indicates a significant difference between this squat condition and all other barbell placements on a p ≤ 0.05 level*.

† *Indicates a significant difference between this squat condition and all other stance widths on a p ≤ 0.05 level*.

‡ *Indicates a significant difference between this squat condition and all other squat conditions a p ≤ 0.05 level*.

**Figure 1:**
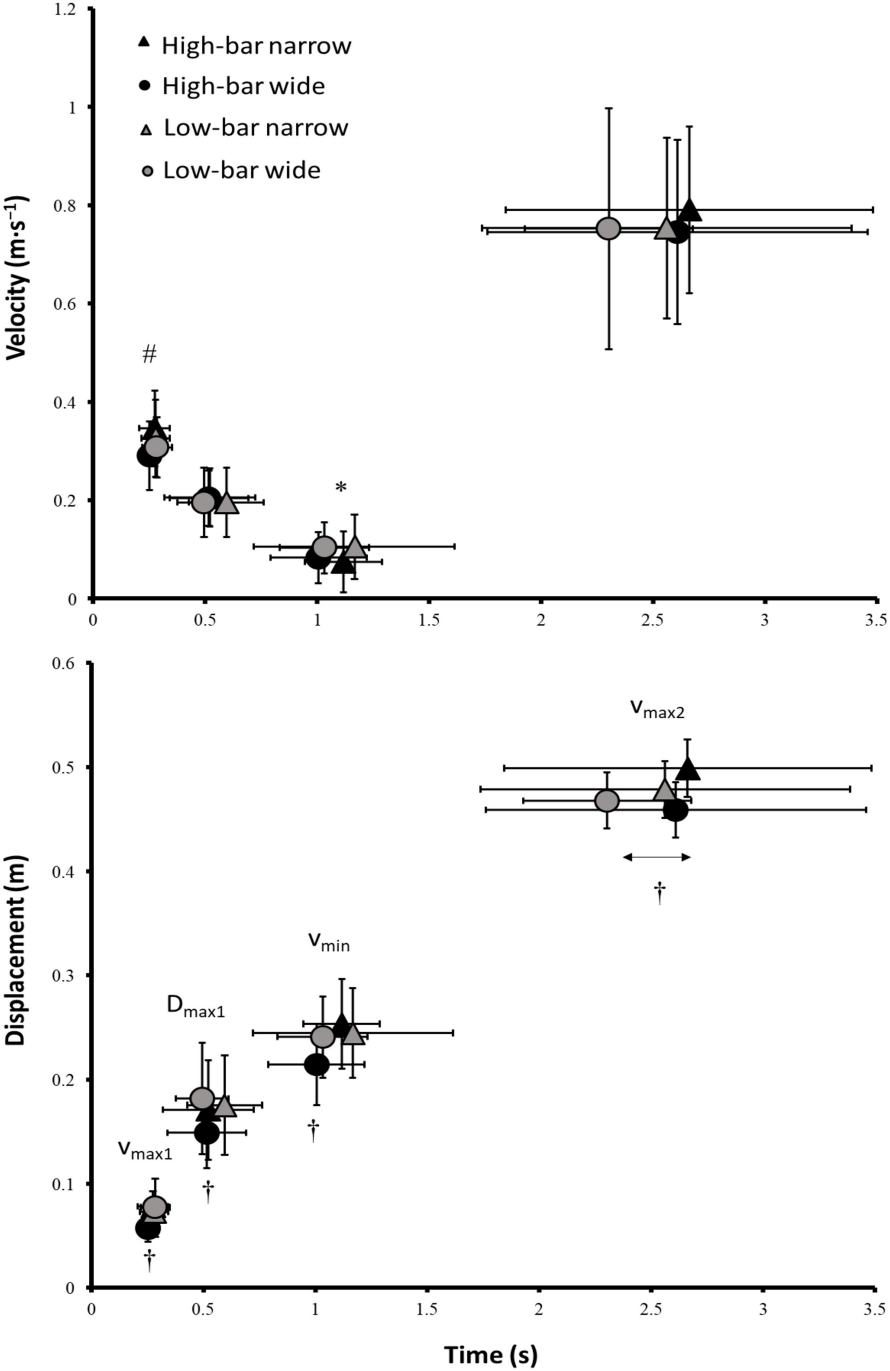
Mean ± SD velocity and displacement of the events *v*_max1_, *d*_max1_, *v*_min_, and *v*_max2_, and their timing. # Indicates a significant difference in velocity between the narrow and wide stance widths on a *p* ≤ 0.05 level. * Indicates a significant difference in velocity between HBNS and LBNS on a *p* ≤ 0.05 level. † Indicates a significant difference in displacement between the HBWS and all other squat conditions on a *p* ≤ 0.05 level. ↔ Indicates a significant difference in timing between the high-bar and low-bar barbell placements on a *p* ≤ 0.05 level.

For the occurrence and timing of the events, a significant effect was found for barbell placement at *v*_max2_ (*F* = 5.8, *p* = 0.035, η^2^ = 0.34; see Figure 1). *Post-hoc* tests revealed that *v*_max2_ occurred earlier for the low-bar conditions (*p* = 0.035). Also, a significant interaction effect for barbell placement with stance width and event was found at every event (*F* ≥ 4.8, *p* ≤ 0.05, η^2^ ≥ 0.30) for barbell displacement from *v*_0_, where the HBWS condition occurred at a lesser displacement compared with all the other squat conditions. A significant effect of stance width upon velocity (*F* = 11.67, *p* = 0.003, η^2^ = 0.37) was found in *v*_max1_ together with a significant effect (*F* = 5.43, *p* = 0.03, η^2^ = 0.21) of barbell placement in *v*_min_. *Post-hoc* tests showed that barbell velocity was greater in *v*_max1_ for the narrow stance widths compared to the wide stance widths (*p* = 0.003). At the same time, in *v*_min_, barbell velocity was greater when squatting with a low-bar condition compared with the high-bar conditions (*p* = 0.03; see Figure 1).

A significant effect of event, stance width, and barbell placement was found for torso angle (*F* ≥ 8.09, *p* ≤ 0.036, η^2^ ≥ 0.62; see Table 3). *Post-hoc* tests revealed that forward lean increased from *v*_0_ and *v*_max1_ to *d*_max1_ and *v*_min_, respectively, before decreasing in *v*_max2_ for all squat conditions. Furthermore, squatting with a low-bar technique resulted in a greater horizontal torso angle than squatting with a high-bar technique (*p* = 0.003) in all events.

A significant interaction effect between event, barbell placement, and stance width was found for hip flexion and hip abduction angles (*F* ≥ 5.8, *p* ≤ 0.005, η^2^ ≥ 0.35). Here *v*_max2_ occurred at a greater hip flexion angle for the low-bar conditions compared with the high-bar conditions (*p* = 0.003; see Table 3). *Post-hoc* tests also showed that greater hip abduction angles were created at *v*_0_, which decreased to *v*_max1_ before remaining stable in the three last events (*p* ≥ 0.116). Furthermore, greater hip abduction angles were observed in all events for the wide stance width for the high-bar placement (*p* ≤ 0.034). Also, a significant interaction effect between event, stance width, and barbell placement occurred for hip internal rotation angles (*F* ≥ 5.4, *p* ≤ 0.012, η^2^ ≥ 0.33). Hip internal angles decreased from *v*_0_ to *d*_max1_ and *v*_min_ before changing to hip external rotation angles in *v*_max2_. Furthermore, squatting with a wide stance width created a greater hip internal rotation angle at *v*_0_, *v*_max1_, *d*_max1_, and *v*_min_ than the narrow stance widths.

Also, a significant interaction effect for event, barbell placement, and stance width was found for knee flexion and plantar flexion angles (*F* ≥ 2.72, *p* ≤ 0.041, η^2^ ≥ 0.20), where squatting with a narrow stance width resulted in greater knee flexion and plantar flexion angles.

**Figure 2:**
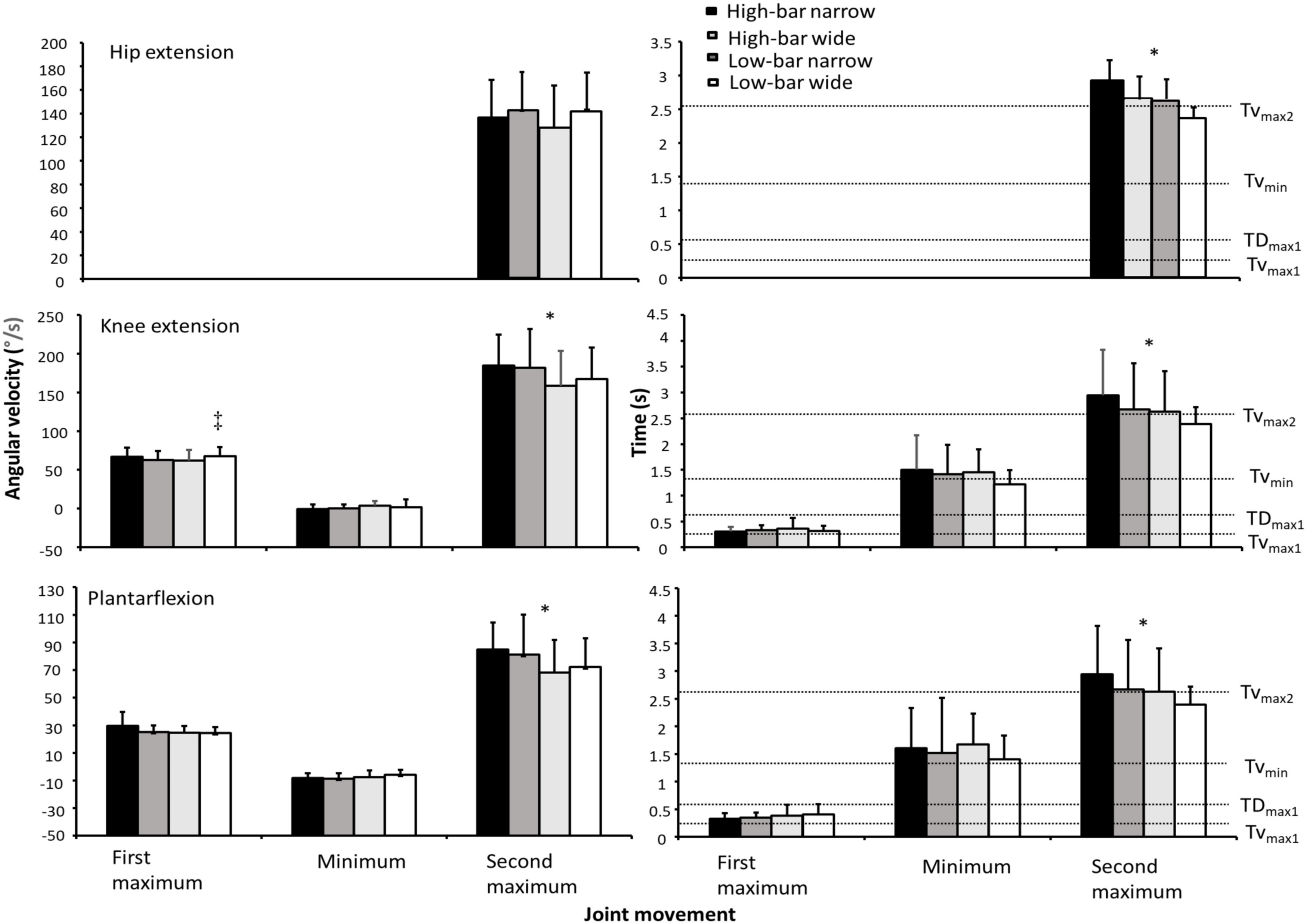
Mean ± SD joint movements at the hip, knee, and ankle joint together with their timings for the HBNS, HBWS, LBNS, and LBWS during 3-RM back squats. Also mean timing of the events *v*_0_, *v*_max1_, *d*_max1_, *v*_min_, and *v*_max2_ for all squat conditions relative to the timing of the joint movements. * Indicates a significant difference between high-bar and low-bar barbell placements for this event on a *p* ≤ 0.05 level. ‡ Indicates a significant difference between this squat condition and all other squat conditions for this event on a *p* ≤ 0.05 level.

The knee and ankle reached peak angular velocity at two distinct points, and their velocity decreased between these points. However, peak hip angular velocity occurred only once (see Figure 2). No significant effects of squat condition were found for hip extension velocity (*F* = 0.51, *p* = 0.489, η^2^ = 0.05), whereas a significant interaction between barbell placement and stance width occurred at the first maximum knee angular velocity and a significant effect of barbell placement at the second maximum knee angular velocity (*F* ≥ 6.2, *p* ≤ 0.03, η^2^ ≥ 0.36). At the first maximum angular velocity, the LBWS showed a greater peak knee extension angular velocity than the other squat conditions. At the second maximum angular velocity, the high-bar conditions produced a greater peak knee extension angular velocity than the low-bar conditions (*p* = 0.03). For the ankle, a significant effect occurred only at the second maximum angular velocity (*F* = 7.5, *p* = 0.019, η^2^ = 0.4), where the high-bar conditions created a greater peak plantar flexion angular velocity than the low-bar conditions (*p* = 0.019). Also, at the second maximum angular velocity, a significant effect of barbell placement was found for every joint (*F* ≥ 5.2, *p* ≤ 0.043, η^2^ ≥ 0.32), where the high-bar conditions produced peak hip, knee, and ankle angular velocities later in the ascent than the low-bar conditions (*p* ≤ 0.043; see Figure 2).

**Figure 3:**
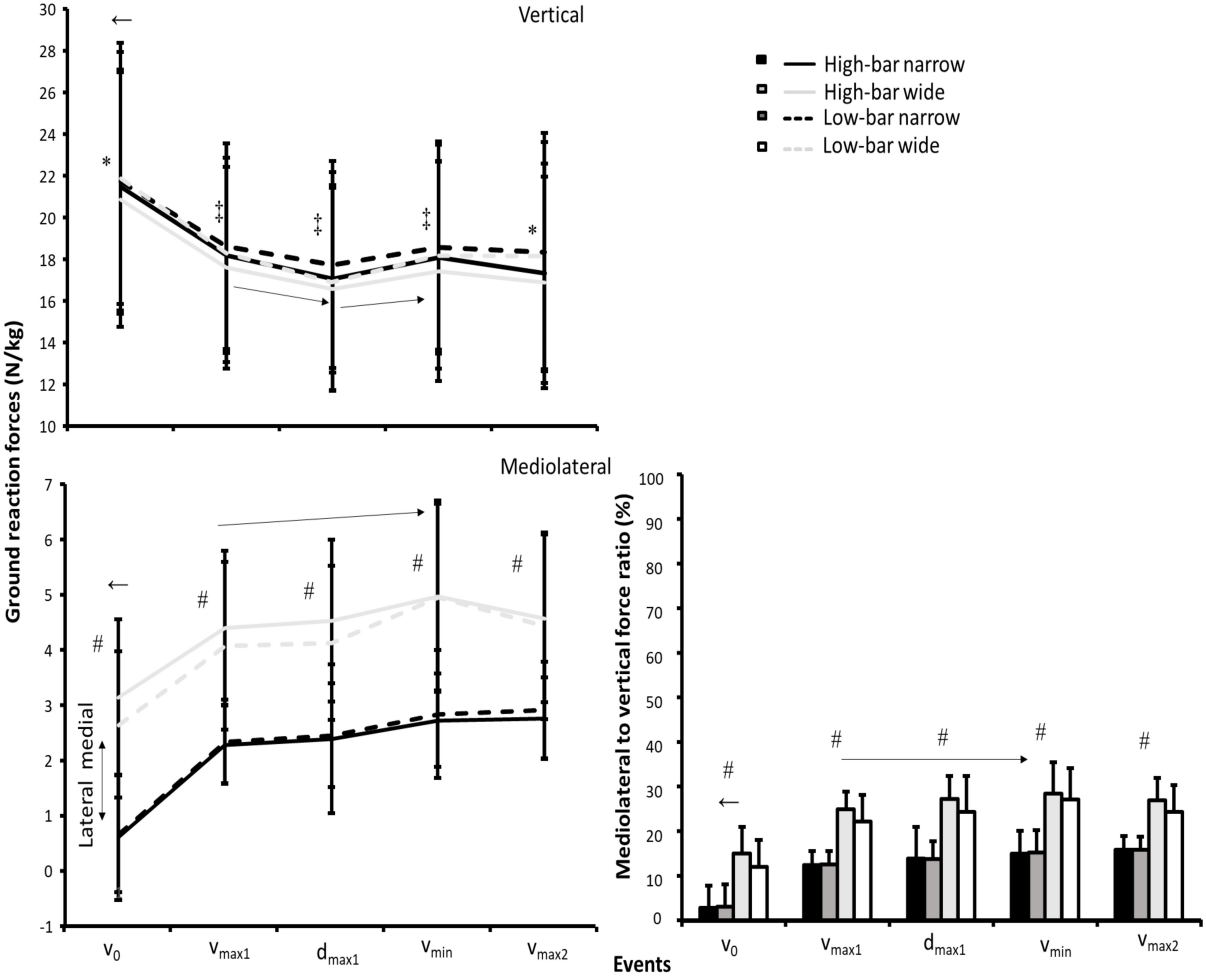
Mean ± SD vertical and mediolateral ground reaction force together with mediolateral to vertical force ratio normalized by body mass for the HBNS, HBWS, LBNS, and LBWS in the events *v*_0_, *v*_max1_, *d*_max1_, *v*_min_, and *v*_max2_ during 3-RM back squats. ← Indicates a significant difference between this event and all other events on a *p* ≤ 0.05 level for each squat condition. * Indicates a significant difference between high-bar and low-bar barbell placements for this event on a *p* ≤ 0.05 level. ‡ Indicates a significant difference between the LBNS squat condition and all other conditions for this event on a *p* ≤ 0.05 level. → Indicates a significant difference between the two events from the start to the end of the arrow on a *p* ≤ 0.05 level for each squat condition. # Indicates a significant difference between the narrow and wide stance widths for this event on a *p* ≤ 0.05 level.

A significant interaction effect was found between event and barbell placement for vertical ground reaction force (*F* ≥ 12.8, *p* ≤ 0.006, η^2^ ≥ 0.59; see Figure 3), whereas the vertical ground reaction forces decreased from *v*_0_ to all other events for all squat conditions (*p* ≤ 0.001). Also, ground reaction forces decreased from *v*_max1_ to *d*_max1_ before increasing in *v*_min_ again for all squat conditions (*p* ≤ 0.014), producing similar ground reaction forces at the events *v*_max1_, *v*_min_, and *v*_max2_. The low-bar conditions produced greater ground reaction forces than the high-bar conditions at *v*_0_ and *v*_max2_, whereas the LBNS resulted in greater vertical ground reaction forces than all other squat conditions at *v*_max1_, *d*_max1_, and *v*_min_ (see Figure 3).

For mediolateral ground reaction forces, a significant effect of event and stance width was found (F ≥ 25.6, *p* ≤ 0.001, η^2^ ≥ 0.72), where the medially-directed ground reaction forces increased from *v*_0_ to all other events and from *v*_max1_ to *v*_min_ (*p* ≤ 0.003). Hence, the wide stance widths created greater medial ground reaction forces than the narrow stance widths (*p* = 0.001). There was a significant interaction effect of event and stance width for the mediolateral/vertical force ratio (*F* ≥ 3.6, *p* ≤ 0.045, η^2^ ≥ 0.27) where the mediolateral to vertical force ratio was greater during all events for the wide stance widths compared with the narrow stance widths (*p* = 0.001). Furthermore, the mediolateral to vertical force ratio increased from *v*_0_ to all events and from *v*_max1_ to *v*_min_ and *v*_max2_ (*p* ≤ 0.014) for all squat conditions.

**Figure 4:**
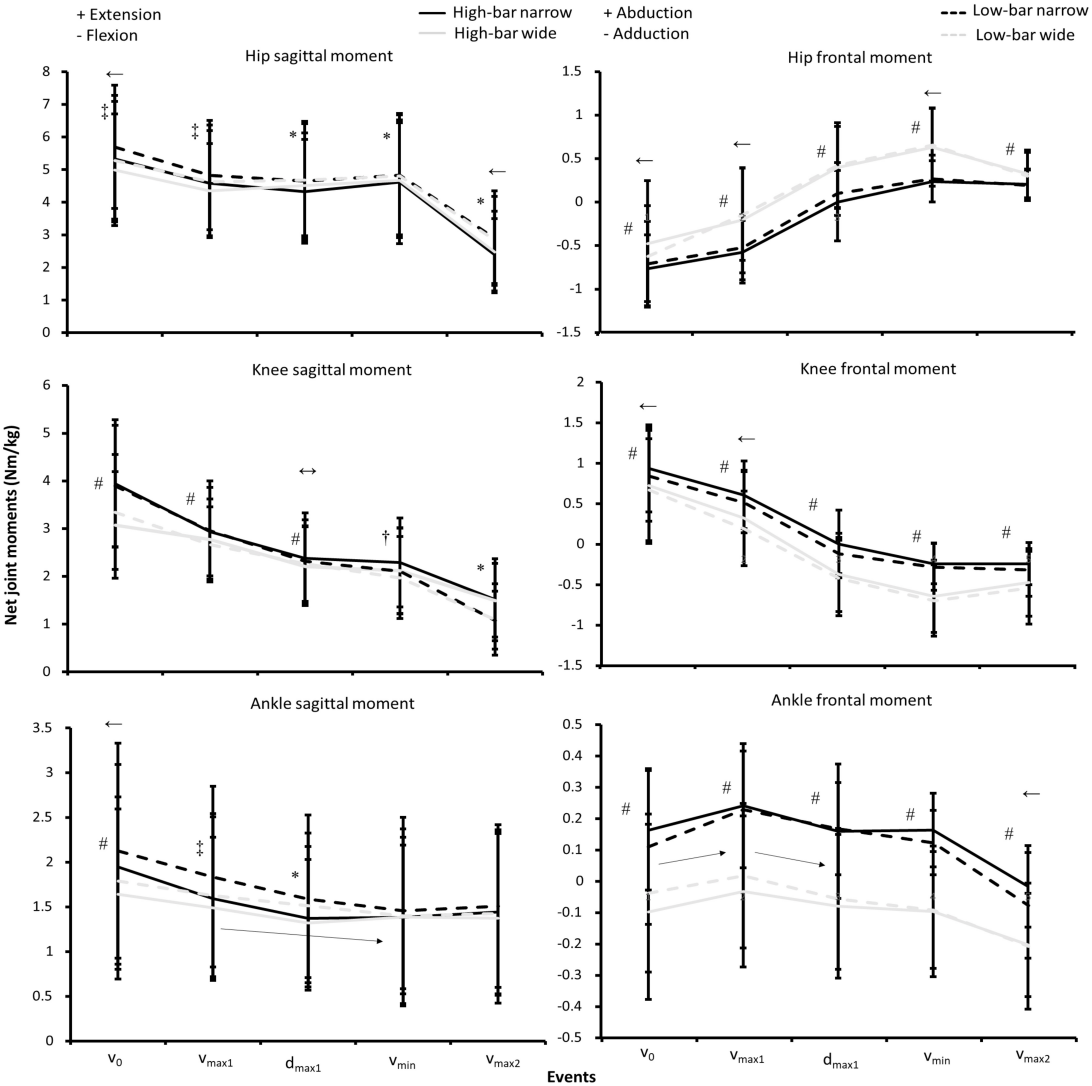
Mean ± SD net joint moments normalized by body mass for the HBNS, HBWS, LBNS, and LBWS in the events *v*_0_, *v*_max1_, *d*_max1_, *v*_min_, and *v*_max2_ during 3-RM back squats. ↔ Indicates a significant difference between all events on a *p* ≤ 0.05 level for each condition. ← Indicates a significant difference between this event and all other events *p* ≤ 0.05 level for each condition. → Indicates a significant difference between the two events from the start to the end of the arrow on a *p* ≤ 0.05 level for each condition. † Indicates a significant difference between the HBNS and all other conditions for this event on a *p* ≤ 0.05 level. * Indicates a significant difference between high-bar and low-bar barbell placements for this event on a *p* ≤ 0.05 level. # Indicates a significant difference between the narrow and wide stance widths for this event on a *p* ≤ 0.05 level. ‡ Indicates a significant difference between the LBNS and all other squat conditions for this event on a *p* ≤ 0.05 level.

Significant interaction effects for event, barbell placement, and stance width were observed (*F* ≥ 3.8, *p* ≤ 0.034, η^2^ ≥ 0.26) for hip extension and plantar flexion moments. Further, a significant interaction effect for event, barbell placement, and stance width was found for knee extension moments (*F* ≥ 3.5, *p* ≤ 0.038, η^2^ ≥ 0.18; see Figure 4).

The Bonferroni *post-hoc* tests revealed that the hip and knee extension, together with plantarflexion moments, decreased from *v*_0_ to all other events (*p* ≤ 0.048). Hip extension moments were stable in *v*_max1_, *d*_max1_, and *v*_min_ before decreasing in *v*_max2_ (*p* = 0.001). Additionally, squatting with a LBNS created a greater hip extension moment in all events compared to the high-bar conditions, where the LBWS demonstrated a greater hip extension moment than the high-bar conditions in *d*_max1_, *v*_min_, and *v*_max2_. Squatting with a narrow stance created the greatest knee extension moments in *v*_0_, *v*_max1_, and *d*_max1_, but the knee extension moments during *v*_min_ and *v*_max2_ were significantly lower during LBNS squats compared with the other squat conditions.

For the hip, knee, and ankle frontal plane moments, a significant interaction effect between event and stance width was found (*F* ≥ 5.4, *p* ≤ 0.007, η^2^ ≥ 0.33; see Figure 4), where hip adduction moments were created in *v*_0_ and *v*_max1_, which changed to hip abduction moments in *d*_max1_, *v*_min_, and *v*_max2_ for all squat conditions. Furthermore, knee abduction moments were created in *v*_0_ and *v*_max1_, which changed to knee adduction moments in *d*_max1_, *v*_min_, and *v*_max2_ for all squat conditions. Greater knee abduction moments were created in *v*_0_ and *v*_max1_ for the narrow squat conditions, whereas greater knee adduction moments were created for the wide stance widths in *d*_max1_, *v*_min_, and *v*_max2_. Also, squatting with a narrow stance width created ankle abduction moments, whereas squatting with a wide stance width created ankle adduction moments during all events.

**Figure 5:**
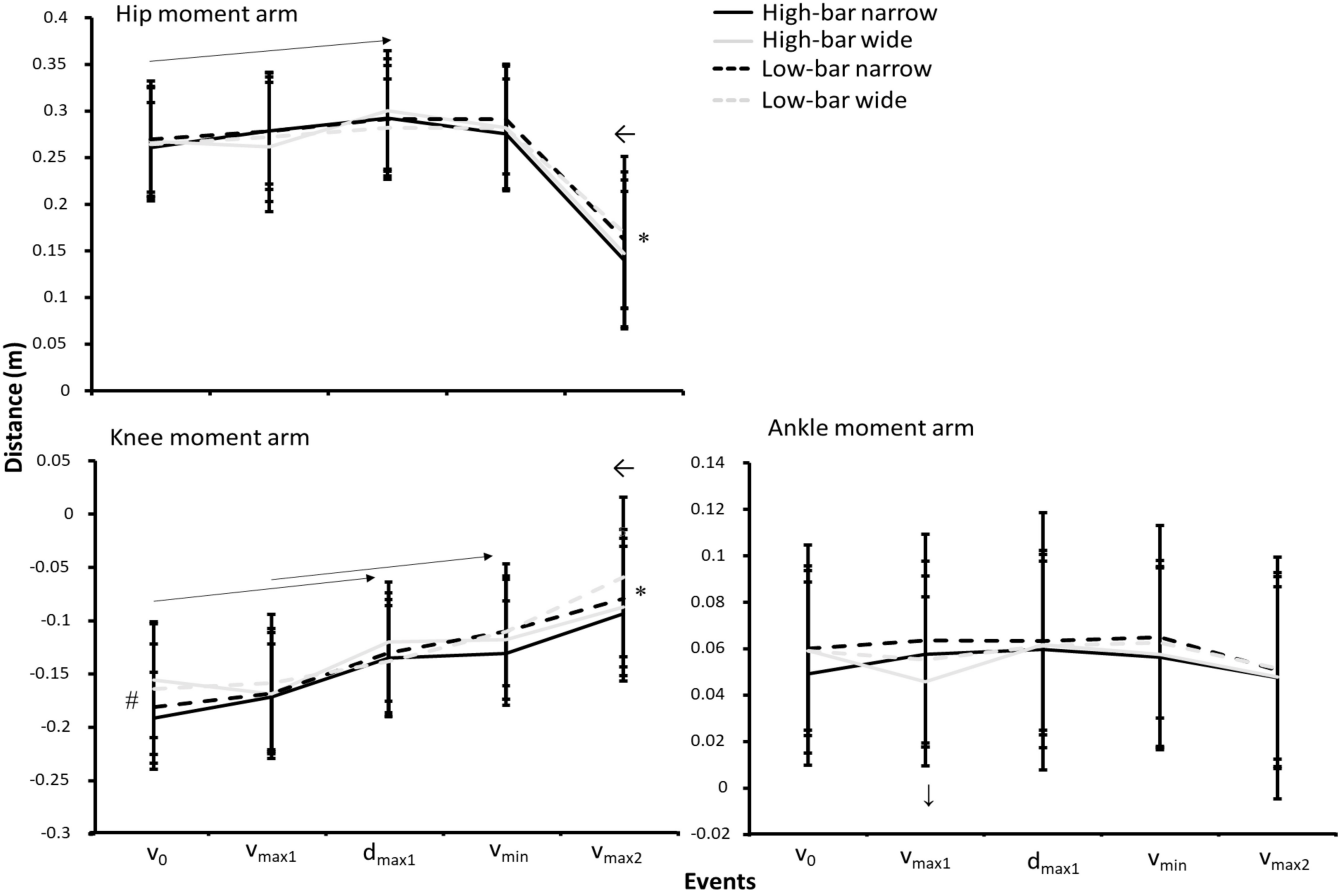
Mean ± SD sagittal moments arms between the hip, knee, and ankle joints and the ground reaction force vector for the HBNS, HBWS, LBNS, and LBWS in the events *v*_0_, *v*_max1_, *d*_max1_, *v*_min_, and *v*_max2_ during 3-RM back squats. ← Indicates a significant difference between this event and all other events on a *p* ≤ 0.05 level for each squat condition. → Indicates a significant difference between the two events from the start to the end of the arrow on a *p* ≤ 0.05 level for each squat condition. * Indicates a significant difference between high-bar and low-bar barbell placements for this event on a *p* ≤ 0.05 level. # Indicates a significant difference between the narrow and wide stance widths for this event on a *p* ≤ 0.05 level. ↓ Indicates a significant difference between the HBWS and all other squat conditions for this event on a *p* ≤ 0.05 level.

Significant interaction effects for event and barbell placement were found for hip and knee moment arm ground reaction forces (*F* ≥ 2.8, *p* ≤ 0.035, η^2^ ≥ 0.19). Additionally, a significant interaction effect for stance width and event was found for the ankle moment arm (*F* = 3.2, *p* = 0.022, η^2^ ≥ 0.22). *Post-hoc* tests revealed that the hip moment arm increased from *v*_0_ to *d*_max1_ and *v*_min_ before decreasing in *v*_max2_ (*p* ≤ 0.025) for all squat conditions. Similar moment arms were produced at the first four events between conditions, however, the low-bar conditions demonstrated greater moment arms at *v*_max2_ than the high-bar conditions (see Figure 5).

Knee moment arms decreased significantly at each event, except between *v*_0_ and *v*_max1_, together with *d*_max1_ and *v*_min_. However, squatting with a narrow stance width showed greater knee moment arms in *v*_0_ before decreasing in the subsequent events. Also, the HBWS knee moment arm increased from *v*_0_ to *v*_max1_, whereas knee moment arms decreased between these events for the other squat conditions reflected by the barbell placement, stance width, and event interaction. Greater knee moment arms were demonstrated at *v*_max2_ during high-bar conditions than low-bar conditions.

**Figure 6:**
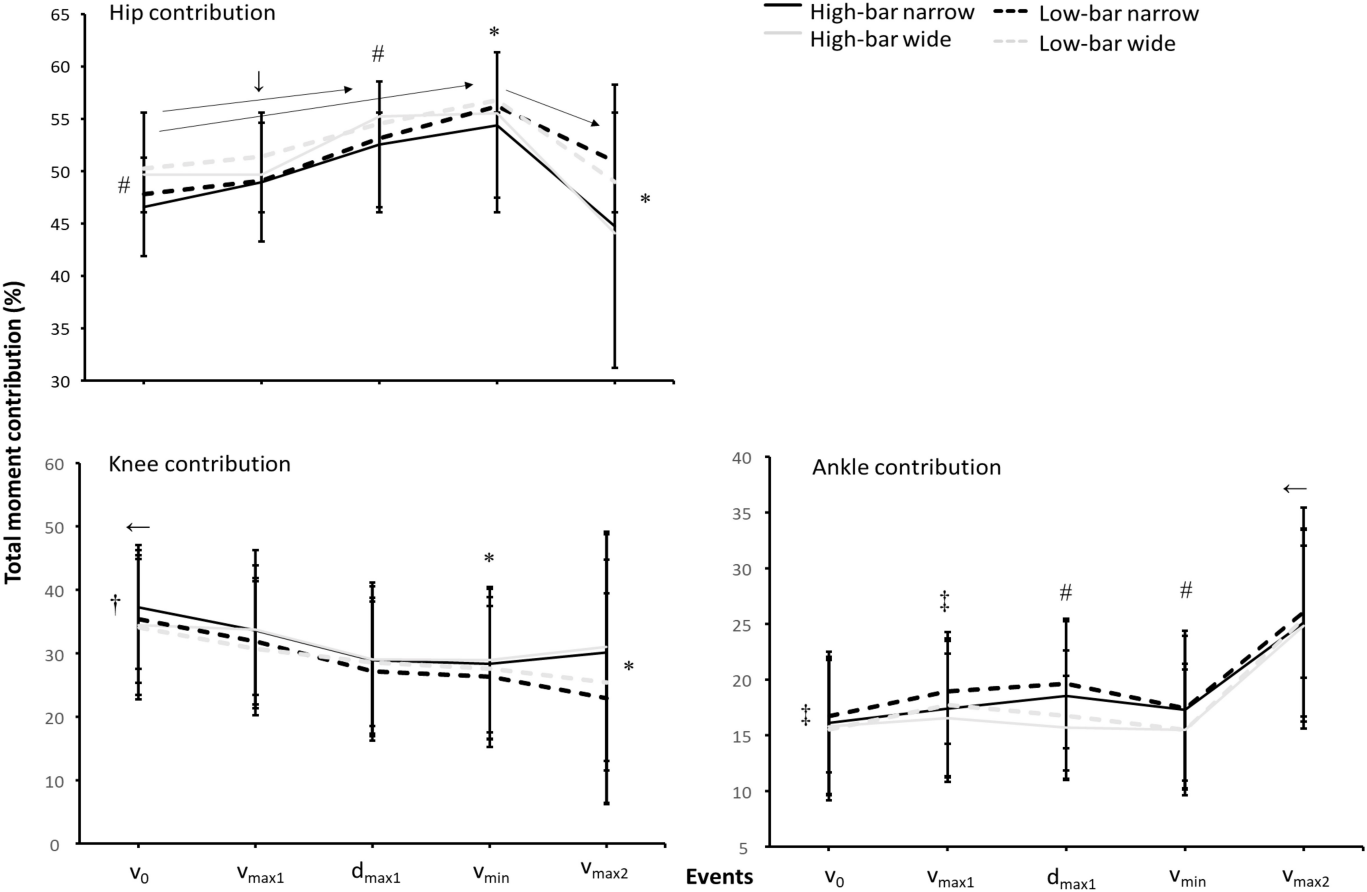
Mean ± SD total moment contributions for the hip, knee, and ankle joints when both frontal and sagittal are calculated for the HBNS, HBWS, LBNS, and LBWS in the events *v*_0_, *v*_max1_, *d*_max1_, *v*_min_, and *v*_max2_ during 3-RM back squats. ← Indicates a significant difference between this event and all other events on a *p* ≤ 0.05 level. → Indicates a significant difference between these two events on a *p* ≤ 0.05 level. # Indicates a significant difference between wide and narrow stance widths for this event on a *p* ≤ 0.05 level. † Indicates a significant difference between the HBNS and all other conditions for this event on a *p* ≤ 0.05 level. * Indicates a significant difference between high-bar and low-bar barbell placements for this event on a *p* ≤ 0.05 level. ↓ Indicates a significant difference between the LBWS and all other conditions for this event on a *p* ≤ 0.05 level. ‡Indicates a significant difference between the LBNS and all other conditions for this event on a *p* ≤ 0.05 level.

For the total moment contribution, a significant effect of event, barbell placement, and stance width was found for the hip knee and ankle joints (*F* ≥ 2.8, *p* ≤ 0.043, η^2^ ≥ 0.24; see Figure 6).

*Post-hoc* tests showed that hip contribution increased from *v*_0_ to *d*_max1_ and *v*_min_ before decreasing at *v*_max2_ for all squat conditions (*p* ≤ 0.023). Furthermore, the LBWS had the greatest hip contribution to the total moment at *v*_0_, *v*_max1_, and *d*_max1_, before *v*_min_ and *v*_max2_, where the LBNS showed a similar hip contribution. Also, squatting with an HBNS produced lower hip contributions during all events compared with the wide stance widths_._ Knee contribution decreased from *v*_0_ to all other events for all squat conditions (*p* ≤ 0.024). The HBNS had a greater knee contribution than all other squat conditions in *v*_0_ while similar knee contributions were produced at *v*_max1_ and *d*_max1_. At *v*_min_ and *v*_max2_, both high-bar placements had a greater knee contribution than the low-bar placements. Ankle contributions were stable throughout the four first events before increasing at *v*_max2_ (*p* ≤ 0.001), where the LBNS had a greater ankle contribution at *v*_0_ and *v*_max1_ than all other squat conditions. The narrow stance width had a greater ankle contribution at *d*_max1_ and *v*_min_ than the wide stance widths before decreasing at *v*_max2_.

**Figure 7:**
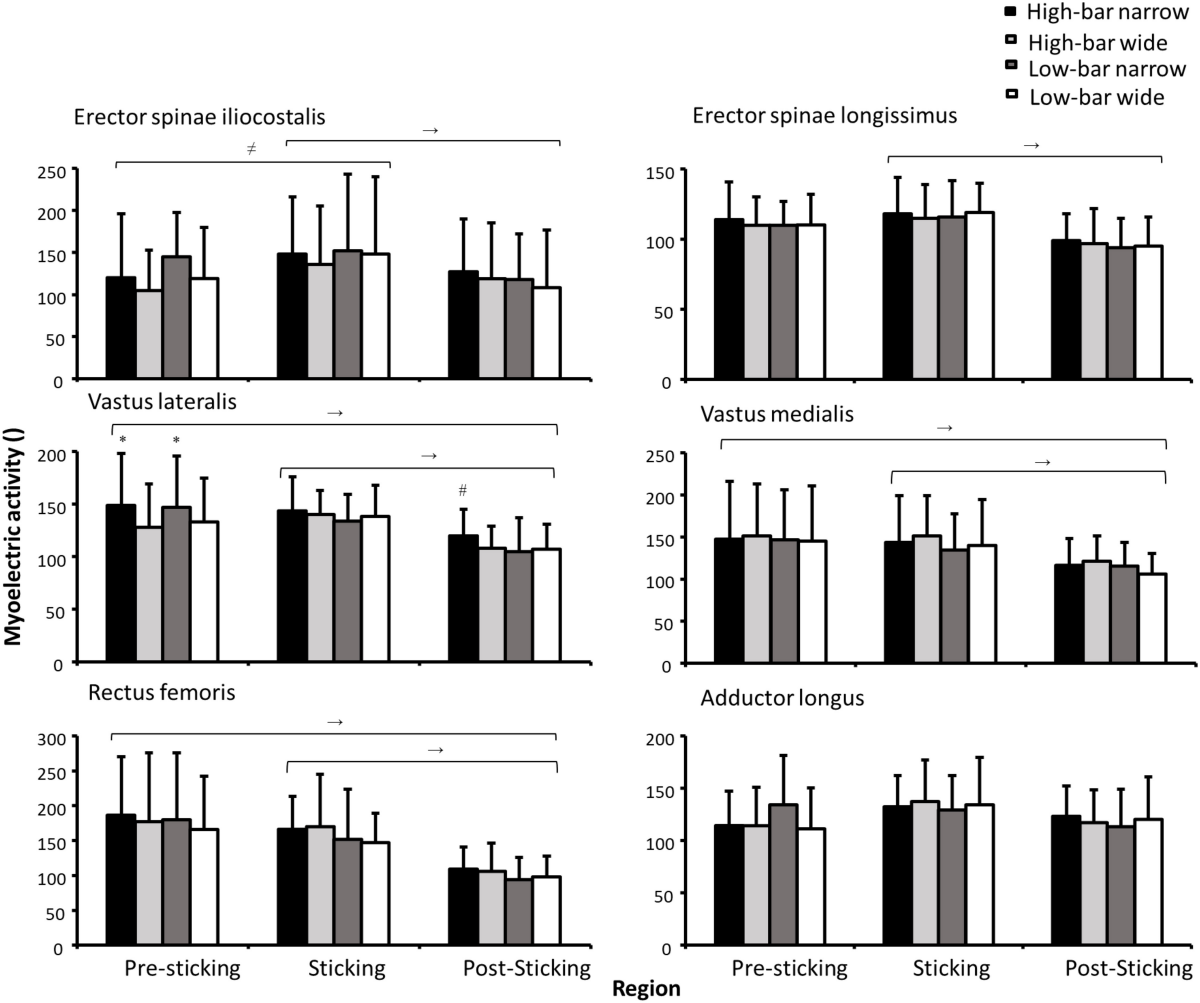
Mean ± SD normalized myoelectric activity for the erector spinae iliocostalis and longissimus, vastus lateralis and medialis, rectus femoris, and adductor longus during 3-RM HBNS, HBWS, LBNS, and LBWS in the presticking, sticking, and poststicking regions. → Indicates a significant difference between these two regions for all conditions on a *p* ≤ 0.05 level. ≠ Indicates a significant difference for the HBNS, HBWS, and LBWS between these two regions on a *p* ≤ 0.05 level. * Indicates a significant difference between this condition and all other stance widths for this region on a *p* ≤ 0.05 level. # Indicates a significant difference between this condition and all other squat conditions in this region on a *p* ≤ 0.05 level.

**Figure 8:**
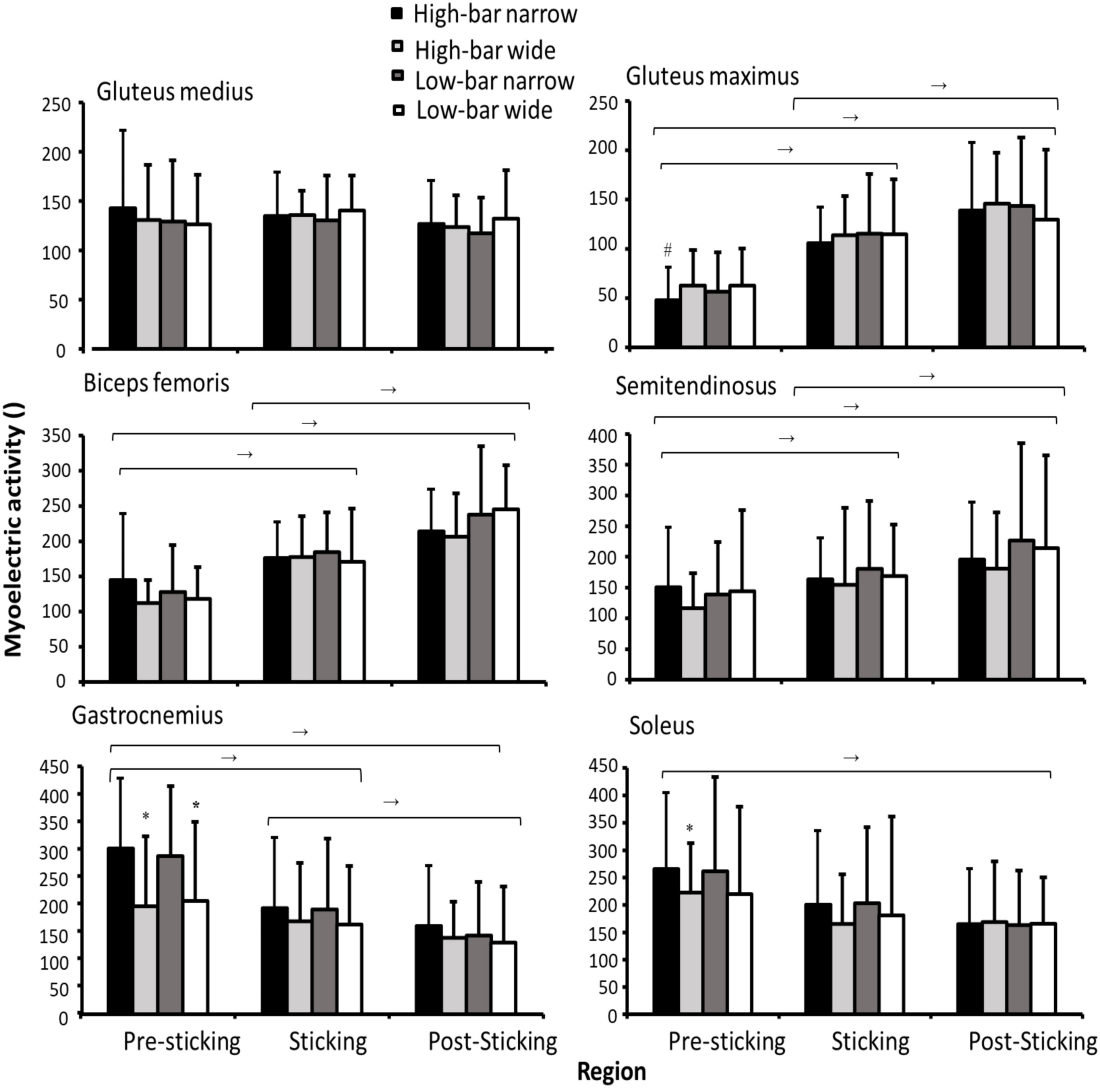
Mean ± SD normalized myoelectric activity for the gluteus medius and maximus, biceps femoris, semitendinosus, gastrocnemius, and soleus during 3-RM HBNS, HBWS, LBNS, and LBWS in the presticking, sticking, and poststicking regions. → Indicates a significant difference between these two regions for all conditions on a *p* ≤ 0.05 level. * Indicates a significant difference between this condition with other stance widths for this region on a *p* ≤ 0.05 level. * Indicates a significant difference between the HBNS and all other conditions for this region on a *p* ≤ 0.05 level.

The myoelectric activity was significantly different in the sticking region for all muscles (*F* ≥ 12.93, *p* ≤ 0.002, η^2^ ≥ 0.46) except for the adductor longus and gluteus medius (*F* ≤ 1.58, *p* ≥ 0.218, η^2^ ≤ 0.067). A significant effect of stance width was found for vastus lateralis activity (*F* = 4.9, *p* = 0.038, η^2^ = 0.20). Also, a significant interaction between barbell placement and stance width was observed for vastus lateralis and gluteus maximus (*F* ≥ 4.62, *p* ≤ 0.044, η^2^ ≥ 0.19). Finally, a significant interaction between the sticking region and the stance width occurred for soleus and gastrocnemius activity (*F* ≥ 4.62, *p* ≤ 0.041, η^2^ ≥ 0.24; see Figures 7, 8).

*Post-hoc* tests showed that myoelectric activity for the erector spinae decreased from the sticking region to the poststicking region for all squat conditions (*p* = 0.011), whereas the myoelectric activity of quadriceps decreased from the presticking and sticking region to the poststicking region for all squat conditions (*p* ≤ 0.025). Furthermore, the narrow stance widths resulted in greater vastus lateralis myoelectric activity than the wide stance widths during the presticking region. A barbell placement stance width interaction effect indicated that the HBNS demonstrated greater vastus lateralis myoelectric activity during the poststicking region compared with all other squat conditions (*p* = 0.044). Squatting with a narrow stance width produced greater gastrocnemius and soleus myoelectric activity in the presticking region. In the sticking and poststicking regions, gastrocnemius and soleus myoelectric activity decreased, as indicated by the sticking point region stance width significant interaction effect. However, the opposite pattern was observed for the gluteus maximus and hamstring muscles, as their activity increased significantly during each sticking point region (*p* ≤ 0.038). Finally, the HBNS squat produced less gluteus maximus myoelectric activity in the presticking region compared with all the other squat conditions, indicated by the significant barbell placement stance width interaction effect.

## Discussion

The main findings from this study were that the sticking region started at a lower barbell height for the HBWS compared with the other squat conditions. Squatting with an HBNS resulted in deeper knee flexion angles than the other squat conditions, and therefore greater squat depth, whereas the low-bar conditions resulted in larger torso inclination at all events. All squat conditions resulted in medially directed ground reaction forces, but to a greater extent with the wide stance width, which increased for all squat conditions in *v*_min_. Furthermore, the knee moment arm decreased during all events, whereas the hip moment arm peaked in *d*_max1_ and *v*_min_, independent of squat condition. Also, the hip joint was responsible for over 50% of the total moment contributions at *d*_max1_ and *v*_min_ for all squat conditions. This finding confirms our hypothesis that the large hip demands around the sticking region may be the main limitation for overcoming it, independent of squat condition. Squatting with an LBWS resulted in greater hip contributions than the other squat conditions, partly confirming our second hypothesis that the low-bar conditions would produce greater hip contributions than the high-bar conditions. Finally, between the squat conditions, vastus lateralis and gastrocnemius myoelectric activity were greater for the narrow stance widths than the wide stance width, whereas gluteus maximus activity was lower for the HBNS than the other squat conditions.

Our findings showed that peak moments were produced at *v*_0_, whereas in the sticking region it started at around 0.15–0.17 m barbell height and at 0.25–0.27 s. This may be explained by muscle potentiation of the quadriceps, caused by the stretch-shortening cycle, which makes it possible to produce more force during the presticking region, as observed in the study of van den Tillaar et al. (2021). The potentiation effect, has in previous studies, been reported to dimmish after around 0.3 s, which is around where the sticking region started for all squat conditions.

Furthermore, the concentric ascent starts with both knee extension and plantar flexion, and there was decreased myoelectric activity from the presticking and sticking to the poststicking region in the quadriceps and plantar flexors for all squat conditions. Therefore, strengthening these muscles should not be neglected, as their greater contribution could result in *d*_max1_ occurring at a higher vertical barbell height, increasing the chance of a successful lift. Moreover, because ground reaction force was at its lowest in *d*_max1_ due to peak deacceleration, it demonstrates that this is the event in the lift where the capability of the lifter to produce force is at its lowest, independent of squat condition. Therefore, we suggest that authors avoid characterizing *v*_min_ (when velocity is lowest) as the sticking point and instead, refer to *d*_max1_ (where deacceleration is highest) as the sticking point, independent of the squat type or stance.

The participants squatted the greatest load with the LBNS followed by the HBNS, the LBWS, and then the HBWS. When squatting with an HBWS, the sticking region started and ended at around 0.03–0.04 m lower than the barbell height of the other squat conditions (Figure 1). Moreover, the HBWS increased forward lean by 2.4° from *v*_0_ to *v*_max1_, whereas the other conditions increased forward lean by 4.3°-4.4° at the same events. This reflected a different development from *v*_0_ to *v*_max1_ for the hip, knee, and ankle moment arms (Figure 5), where the hip and ankle moment arm decreased and the knee moment arm increased for the HBWS whereas all other squat conditions showed the opposite pattern. It is speculated that the combination of the lower barbell height for the HBWS from *v*_0_ compared with the other squat conditions, together with a smaller forward lean at the start of the sticking region, reduces the contribution of the hip extensors to the hip extensor moment. This may be because an increased forward lean lengthens the hip extensors, increasing the ability of these muscles to generate the force (Escamilla et al., 2001).

Furthermore, squatting with an LBNS resulted in the participants lifting >3 kg greater loads than the other squat conditions. This was in contrast with the findings reported by Lahti et al. (2019), who reported no significant differences in the load lifted between stance widths. This may be explained by two factors. First, squatting with an LBNS resulted in a greater hip extension moment at *v*_0_ and *v*_max1_ than the other squat conditions. It may be that putting greater demands on the hip extensors at the beginning of the ascent could be advantageous when the goal is to lift the greatest loads possible because the myoelectric activity of the quadriceps has been reported to not change much between >50 and 90% of 1-RM (van den Tillaar et al., 2019), implying that the quadriceps may already be near-maximal activation at lower percentages of 1-RM. Therefore, choosing a technique that places more demand on hip extension moments could enable greater loads to be lifted. Second, participants in the study of Lahti et al. (2019) squatted with ~1.0 and 1.5 times greater trochanter width for the narrow and wide stance widths. The participants in the current study squatted with 0.7 and 1.7 shoulder width, resulting in the mediolateral to vertical force ratio being ~50% higher for the wide stance widths compared with the narrow stance widths, and thereby, producing 1.5 to 2 times higher laterally-directed forces for the wide stance widths than the narrow stance widths (Figure 3). This created a superior medially-directed resultant force vector and thereby, greater hip abduction together with knee and ankle adduction moments for the wide stance widths. More importantly, our data indicate that squatting with 1.7 times shoulder width results in around 50% greater laterally-directed forces exerted against the ground for the wide stance width. Moreover, the larger hip abduction moment for the wide stance widths resulted in greater hip contributions to the total moment for the wide stance widths, and especially the LBWS. The larger hip contributions were a result of lower knee extension and plantar flexion moments during the presticking region together with greater hip abduction moments in the sticking region for the wide stance widths (Figure 4). It is speculated that the larger hip abduction and knee adduction moments together with the greater mediolateral to vertical force ratios around the sticking region for the wide stance widths resulted in a less effective vertical lifting technique, despite greater hip contributions compared with the narrow stance widths. Therefore, our findings suggest that the LBWS, typically referred to as a hip dominant squat condition in the literature (Glassbrook et al., 2017), enables greater hip contributions to the total extensor moment in the sticking region because of the hip abduction moments.

The vastus lateralis produced greater myoelectric activity during the narrow stance widths and during the post-sticking region for the HBNS compared with all other squat conditions. Since the HBNS demonstrated 5°-7° greater peak knee flexion than the wide stance widths together with 3°-4° greater flexion at the start of the post-sticking region, the increased knee flexion angle during these regions probably caused increased mechanical work and thereby increased vastus lateralis myoelectric activity. This is supported by Bryanton et al. (2012) who investigated both squat depth and barbell load on the relative hip, knee, and ankle muscular effort between 50 and 90% of 1-RM in back squats and found that the knee extensor relative muscle effort (the ratio of net joint moment to maximum voluntary torque, matched for joint angle) increased with deeper knee flexion angles, but not barbell load. The HBNS produced less gluteus maximus activity in the presticking region compared with other squat conditions, which is logical since increased depth lengthens the gluteus and reduces its capability to produce force (Vigotsky and Bryanton, 2016). However, no significant differences were observed in myoelectric activity for the vastus medialis. It is speculated that the vastus medialis was maximally activated for all squat conditions, and during the wide stances, the inability to produce additional myoelectric activity resulted in greater hip internal rotation and, thereby, a greater knee valgus for the wide stance widths. For the shank, plantar flexion moments were greater while performing back squats with a narrow stance width during the presticking region (Figure 4). This occurred since ankle flexion angles were 4°-9° greater during all events for the narrow stance widths and were not influenced much by barbell placement. Similar findings were reported by Swinton et al. (2012), who found that peak ankle flexion was ~10° greater for narrow stance widths, whereas the present study reported 5°-7° greater ankle flexion for the narrow stance.

Furthermore, larger spinal erector myoelectric activity was observed in the sticking region compared with the poststicking region for all squat conditions. Also, greater myoelectric activity was observed in the presticking region compared with the poststicking region for the erector spinae iliocostalis muscle. These muscles are especially important in the squats because they help to maintain anteroposterior spinal integrity, providing a contribution to spinal stabilization (Schoenfeld, 2010). The higher erector spinae myoelectric activity during the sticking region was probably observed because of the increased forward lean at this point of the lift, resulting in greater hip moment arms and increasing the demands for the erector spinae to contribute to spinal stabilization. This finding is in accordance with a previous study that observed increased myoelectric activity when forward lean increased, compared with a neutral position (Zimmermann et al., 1993).

## Limitations and Further Directions

The participants in this study were recreationally trained lifters and not powerlifters or strength athletes. Therefore, our findings may not be generalizable to powerlifters or strength athletes. Also, the present study only reported the net joint forces calculated and resultant moments from inverse dynamics analyses, and not the joint contact forces (Vigotsky et al., 2019). This method neglects muscle forces, which often are the primary sources of joint loading (Vigotsky et al., 2019). Therefore, further research should use musculoskeletal modeling techniques to quantify the joint contact force.

## Conclusion and Practical Application

Squatting with an LBWS produced greater hip contributions to the total moment, whereas squatting with an HBNS resulted in deeper knee flexion angles and higher knee contributions to the total moment, together with less gluteus maximus and higher vastus lateralis myoelectric activity. Therefore, our findings suggest that training with an HBNS could be beneficial when targeting the knee extensors and plantar flexors, whereas an LBWS could be beneficial when targeting the hip extensors.

## Data Availability Statement

The raw data supporting the conclusions of this article will be made available by the authors, without undue reservation.

## Ethics Statement

The studies involving human participants were reviewed and approved by Norwegian Center for Research Data project number 701688. The patients/participants provided their written informed consent to participate in this study.

## Author Contributions

All authors were involved in planning the study, while EK and SL performed the data collection and analysis. SL wrote the first draft, while the other three authors others discussed and rewrote the manuscript. RT was the supervisor of the project. All authors contributed to the article and approved the submitted version.

## Conflict of Interest

The authors declare that the research was conducted in the absence of any commercial or financial relationships that could be construed as a potential conflict of interest.

## Publisher's Note

All claims expressed in this article are solely those of the authors and do not necessarily represent those of their affiliated organizations, or those of the publisher, the editors and the reviewers. Any product that may be evaluated in this article, or claim that may be made by its manufacturer, is not guaranteed or endorsed by the publisher.
